# *SDHD* promoter mutations are rare events in cutaneous melanomas but *SDHD* protein expression is downregulated in advanced cutaneous melanoma

**DOI:** 10.1371/journal.pone.0180392

**Published:** 2017-06-29

**Authors:** Helena Pópulo, Rui Batista, Cristina Sampaio, Joana Pardal, José Manuel Lopes, Paula Soares

**Affiliations:** 1 Institute of Molecular Pathology and Immunology, University of Porto (IPATIMUP), Porto, Portugal; 2 Institute for Research and Innovation in Health, University of Porto, Porto, Portugal (Instituto de Investigação e Inovação em Saúde, Universidade do Porto, Porto, Portugal); 3 Medical Faculty, University of Porto, Porto, Portugal; 4 Department of Pathology, Hospital S. João, Porto, Portugal; 5 Department of Pathology and Oncology, Medical Faculty, University of Porto, Porto, Portugal; University of Alabama at Birmingham, UNITED STATES

## Abstract

**Background:**

SDHD promoter mutations were reported in 4–10% of cutaneous melanomas. The advanced clinico-pathological and patient survival association with SDHD mutation and/or expression in cutaneous melanoma remains controversial.

**Objectives:**

To evaluate the presence of SDHD promoter mutations and SDHD protein expression in a melanoma series and its possible association with prognosis and survival of the patients.

**Methods:**

We assessed SDHD promoter status in cutaneous melanomas (CM), ocular melanomas (OM) and melanoma cell lines, and the expression of SDHD protein by immunohistochemistry in CM and OM, and by western blot in melanoma cell lines. We explored the putative association between SDHD protein expression and clinico-pathological and prognostic parameters of melanoma.

**Results:**

We detected 2% of SDHD promoter mutations in CM, but none in OM and cell lines. SDHD protein expression was present in all CM, in OM and in all CM and OM derived cell lines analysed. A significant association between lower SDHD mean protein expression and presence of ulceration and higher pT stage was found.

**Conclusions:**

SDHD promoter mutation seems to be a rare event in CM but SDHD lower expression might associate with worst prognostic features in CM.

## Introduction

SDHD is one of the four subunits that compose the Succinate Dehydrogenase (SDH) complex [1]. SDH complex has a central role in mitochondrial metabolism, being a component of the tricarboxylic acid cycle (TCA) by catalysing the oxidation of succinate to fumarate, and of the electron transport chain by transferring electrons to ubiquinone [2].

The *SDH* genes act as tumour suppressor genes, showing loss of heterozygosity (LOH) in combination with germline inactivating mutations in several tumours [1]. *SDHD* alterations where reported in sporadic and familial paraganglioma, phaeochromocytoma and gastrointestinal stromal tumour (GIST) [3].

Following the identification of *TERT* promoter mutations in cancer [4–7], several authors have screened other promoter regions searching for mutations that can be relevant in cancer. Promoter mutations in *SDHD* where recently described by Weinhold *et al* in 10% of cutaneous melanoma, based on data mining, using the whole-genome sequences of human tumours collected from The Cancer Genome Atlas and other public sources. The mutations in *SDHD* promoter associated with reduced gene expression and decrease patient survival [8]. Scholz *et al* reported 4% of *SDHD* promoter mutations in a cohort of cutaneous melanomas, but not related with clinico-pathologic factors or patient survival, and no mutations were found in ocular, mucosal and occult melanomas [9].

Cutaneous melanomas (CM) are very aggressive and, although CM represent <5% of all skin cancers, they are responsible for most of skin cancer-related deaths [10]. Most CM are diagnosed in early stage, with a 5-year survival rate reaching 98% [10], but for patients with metastatic melanoma the median survival is 8–9 months [11]. The risk factors of melanoma development include environmental causes (e.g. sunlight/UV exposure) and genetic predisposition, namely fair complexion, red hair and multiple nevi [12]. CM is a very heterogeneous tumour and many cell signalling pathways are deregulated in melanomagenesis [13, 14]. Genetically, CM harbour a high frequency of activating mutations in oncogenes, such as *BRAF*, *NRAS* and *c-KIT*; loss of tumour suppressors, such as *CDKN2A* and *PTEN* [15], and also the recently discovered *TERT* promoter mutations [4, 5, 16].

Ocular melanomas (OM) are the most frequent primary eye tumour in adults, and account for approximately 5% of all melanomas [17]. The aetiology of uveal and conjunctival melanomas remains elusive and the role of sunlight/UV exposure remains controversial [18, 19]. Mutations in genes associated with CM are less frequently reported in OM [20–23]. *BRAF* and *NRAS* mutations were reported in 14–40% and 0–18%, respectively, of conjunctival melanomas [21, 23–26], whereas they seem to be absent in uveal melanomas [20, 22], in which *GNAQ* and *GNA11* activating mutations [27–29] and loss of *BAP1* [30] are prevalent. *TERT* promoter mutations were reported in conjunctival melanomas, ranging from 0 to 32% and, so far, only one case of uveal melanoma harbouring a TERT promoter mutation was reported [7, 31].

In this study, we assessed the presence of *SDHD* promoter mutations and SDHD protein expression in CM, OM and in melanoma cell lines, in which we already determined *BRAF*, *NRAS*, *GNAQ* and *TERT* promoter mutational status [16, 28, 32]. In addition, we evaluated the possible association between SDHD protein expression, prognosis and survival of patients with CM.

## Materials and methods

### Sample selection, clinical-pathological and prognostic parameters

Formalin-fixed, paraffin-embedded tissues from 107 CM and 35 OM (29 uveal and 6 conjunctival melanomas) were retrieved from the Department of Anatomic Pathology of the Hospital S. João, Porto, and of Hospital S. Marcos, Braga. Clinico-pathological (Tables 1 and 2). Follow-up data were obtained from the patients’ records, the Oncology Registries of Hospital S. João and of Hospital S. Marcos, and from RORENO (Oncology Registry of North Region). All cases were revised and staged according to the 7^th^ edition of AJCC [11]. In CM, follow-up data included time of recurrences and metastases (disease-free survival; DFS) (n = 96) and death due to melanoma (overall survival; OS) (n = 105). The mean follow-up time of the patients for DFS was 52 months (SE±3.94, range 1–195) and for OS was 56 months (SE±3.82, range 1–207). This work was approved by the Local Ethical Committee (CES) and was in accordance with the National ethical rules and Helsinki declaration.

**Table 1:** Clinico-pathological features of cutaneous melanomas.

Clinico-pathological features	
Number of cases (n)	107
Median age (range)	61.7 (7–95)
Gender [n (%)]	
Female	61 (57.0)
Male	46 (43.0)
Sun exposure (body site)	
absent	25 (23.6)
intermittent	64 (60.4)
chronic	17 (16.0)
Histological subtype [n (%)]	
LMM	13 (12.1)
ALM	22 (20.6)
NM	18 (16.8)
SSM	54 (50.5)
Pigmentation	
absent	8 (7.9)
present	93 (92.1)
Median thickness (range) [mm]	3.9 (0–70)
Epidermal ulceration [n (%)]	
absent	70 (65.4)
present	37 (34.6)
Clark level (≤ 1mm) [n (%)]	
I	18 (36.0)
II	17 (34.0)
III	14 (28.0)
IV	1 (2.0)
V	0 (0.0)
Mitotic rate [n (%)]	
< 1/mm^2^	36 (33.6)
≥ 1/mm^2^	71 (66.4)
pT [n (%)]	
≤ pT2	54 (50.5)
> pT2 Mutation status wt *BRAFV600* *NRASQ61* *TERT promoter* *BRAFV600/ TERT promoter*	53 (49.5) 56 (52.3) 20 (18.7) 8 (7.5) 11 (10.3) 12 (11.2)

**Table 2:** Clinico-pathological features of ocular melanomas.

Clinico-pathological features	Uveal melanomas	Conjunctival melanomas
Number of cases [n (%)]	29 (82.9.4)	6 (17.1)
Median age (range)	55 (14–90)	63 (28–90)
Cytological type [n (%)]		
epithelioid	5 (17.2)	4 (80.0)
spindle	12 (41.4)	1 (20.0)
mixed	12 (41.4)	0
pT [n (%)]		
≤ pT2	21 (72.4)	5 (83.3)
> pT2	8 (27.6)	1 (16.7)
Mitotic rate [n (%)]		
≤ 1/mm2 > 1/mm2	21 (72.4) 8 (27.6)	3 (60.0) 2 (40.0)
Median thickness [mm (range)]	NA	3 (0.3–7)
Median basal tumour diameter [mm (range)]	10.9 (3–18)	NA
Tumour scleral involvement [n (%)]		
present	8 (27.6)	NA
absent	21 (72.4)	

NA–Not Applicable

### Cell lines and culture conditions

BLM, G361 and Mewo skin melanoma cell lines were kindly provided by Dr. Marc Mareel, from the Department of Radiotherapy and Nuclear Medicine, Ghent University Hospital, Belgium. A375 skin melanoma cell line was kindly provided by Dr. Madalena Pinto, from CEQUIMED, Faculty of Pharmacy, University of Porto, Portugal. 92.1 [33], OCM1 [34], OMM1 [35], OMM2.3 [36], Mel202 [37], Mel270 [36] and Mel285 [37] uveal melanoma cell lines were kindly provided by Dr. Martine Jager, from the Laboratory of Ophthalmology, Leiden University, Netherlands. All the cell lines were tested for mycoplasma.

BLM, and Mewo cell lines were maintained in DMEM medium (Gibco/BRL–Invitrogen), G361 cell line was maintained in McCoy’s medium (Gibco/BRL–Invitrogen), and 92.1, OCM1, OMM1, OMM2.3, Mel202, Mel270 and Mel285 cell lines were maintained in RPMI medium (Gibco/BRL–Invitrogen). All media were supplemented with 10% of fetal bovine serum, 100U/mL Penicillin and 100ug/mL Streptomycin. Cell lines were maintained in a humidified atmosphere (5% CO2) at 37°C.

### DNA extraction

Extraction of DNA from tumours smaller than 5mm was performed after microdissection with PALM MicroLaser Systems (PALM, Germany) and using the Quiamp DNA micro kit (Quiagen, Hilden). In tumours larger than 5mm, DNA extraction was done by manual dissection of 10μm whole sections of paraffin-embedded tissue using the Invisorb spin tissue mini kit (Invitek, Berlin). DNA extraction from the cell lines was also performed with the Invisorb spin tissue mini kit (Invitek, Berlin).

### Mutation analysis

Fragments encompassing *SDHD* promoter region were amplified by polymerase chain reaction (PCR) of the tumour samples with the sets of primers: Fwd: 5’-CTCCGCCATTGTTCGCCTCA-3’, Rev: 5’-TTCCTGAGGGCTCAAGGTCAT-3’. Genomic DNA (25–100 ng) was amplified by PCR using the following cycling conditions: 30s at 94°C, 90s at 59°C and 60s at 72°C for 35 cycles. Products were enzymatically purified and directly sequenced in an ABI Prism 3130 xl Automatic sequencer (Perkin-Elmer, Foster City, CA) using the BigDye Terminator Sequencing kit (Perkin-Elmer). Cases with mutations were confirmed by an independent amplification.

*BRAF*, *NRAS*, *GNAQ* and *TERT* mutational analysis in the series was previously reported [16, 28, 32].

### Immunohistochemical analysis

Paraffin sections were deparaffinised and rehydrated, followed by a microwave antigen retrieval procedure with 10 mM sodium citrate buffer pH 6.0. The sections were incubated overnight at 4°C in a humidified chamber with the primary antibody SDHD (polyclonal, rabbit, 1:100), from Santa Cruz Biotechnology. The antibody was validated by the manufacturer to ensure the antibody specificity to the target protein and IHC procedures were already published [38]. The detectionwas obtained with the alkaline phosphatase method (APAAP), with the EXPOSE Mouse and Rabbit Specific AP (red) Detection IHC Kit (ab94734; Abcam; Cambridge, UK), and the colour was developed with fast red chromogen, or with a streptavidin–biotin immunoperoxidase detection system with the Ultravision Quanto Detection System HRP (Thermo Scientific, Fremont, USA), and the immunohistochemical staining was developed with AEC substrate HIGHDEF® Red IHC (Enzo Life Sciences, Inc., New York, USA). The slides were counterstained with haematoxylin, and then mounted using a water-miscible mounting medium. A pancreatic endocrine tumour case, previously tested, was used as negative (omission of primary antibody) and positive control. pERKs and TERT expression in the series has been previously reported [16, 32].

### Immunohistochemical evaluation

Two observers (J.M.L. and H.P.) evaluated tumour cell immunoreactivity without knowledge of any clinical and mutational data from the cases. An IHC score was settled for SDHD, and results from the multiplication of the intensity of staining (negative = 0, weak = 1, moderate = 2 and strong = 3) and the proportion of cells showing an unequivocal positive reaction (0–5% = 0, 6–25% = 1, 26–50% = 2, 51–75% = 3, 76–100% = 4).

### Western blot analysis and antibodies

Cells were lysed for 15 min at 4°C using RIPA buffer (1% NP-40 in 150 mM NaCl, 50 mM Tris [pH 7.5], 2 mM EDTA) containing phosphatase and protease inhibitors. Proteins were quantified using a modified Bradford assay (Biorad). Protein samples (50 μg) were separated in 10% SDS/PAGE gels and electroblotted to Hybond ECL membrane (Amersham Biosciences). SDHD (polyclonal, rabbit, 1:200, Santa Cruz Biotechnology) was used. Secondary antibody was conjugated with peroxidase (Santa Cruz Biotechnology) and visualized by the ECL detection solution. Membrane was re-stained with a goat polyclonal anti-actin (Santa Cruz Biotechnology) for loading protein control. XTC1, a hurthle cell thyroid cell line, was used as positive control.

### Statistical analysis

Statistical analysis was performed using STAT VIEW-J 5.0 (SAS Institute, Inc., Cary, NC). The relationship between the average expression level (score) of the immunohistochemistry markers and clinical-pathological parameters was evaluated by ANOVA. When appropriate, multiple comparison corrections were performed using the post hoc Bonferroni or Tamhane tests. The correlation between the immunoreactivity score of the different markers was assessed using the Fisher’s exact test. The Kaplan-Meier method and log-rank test were used to evaluate the melanoma survival data. Univariate analyses were performed to determine the prognostic value of covariates regarding OS and DFS using the Cox regression model. OS and DFS were calculated from the time of diagnosis until death due to disease or metastasis, respectively, or censored at the time of the latest follow-up or death unrelated to the disease. A p value <0.05 was considered statistically significant.

## Results

### *SDHD* promoter mutations analysis in CM, OM and melanoma cell lines

**Fig 1:**
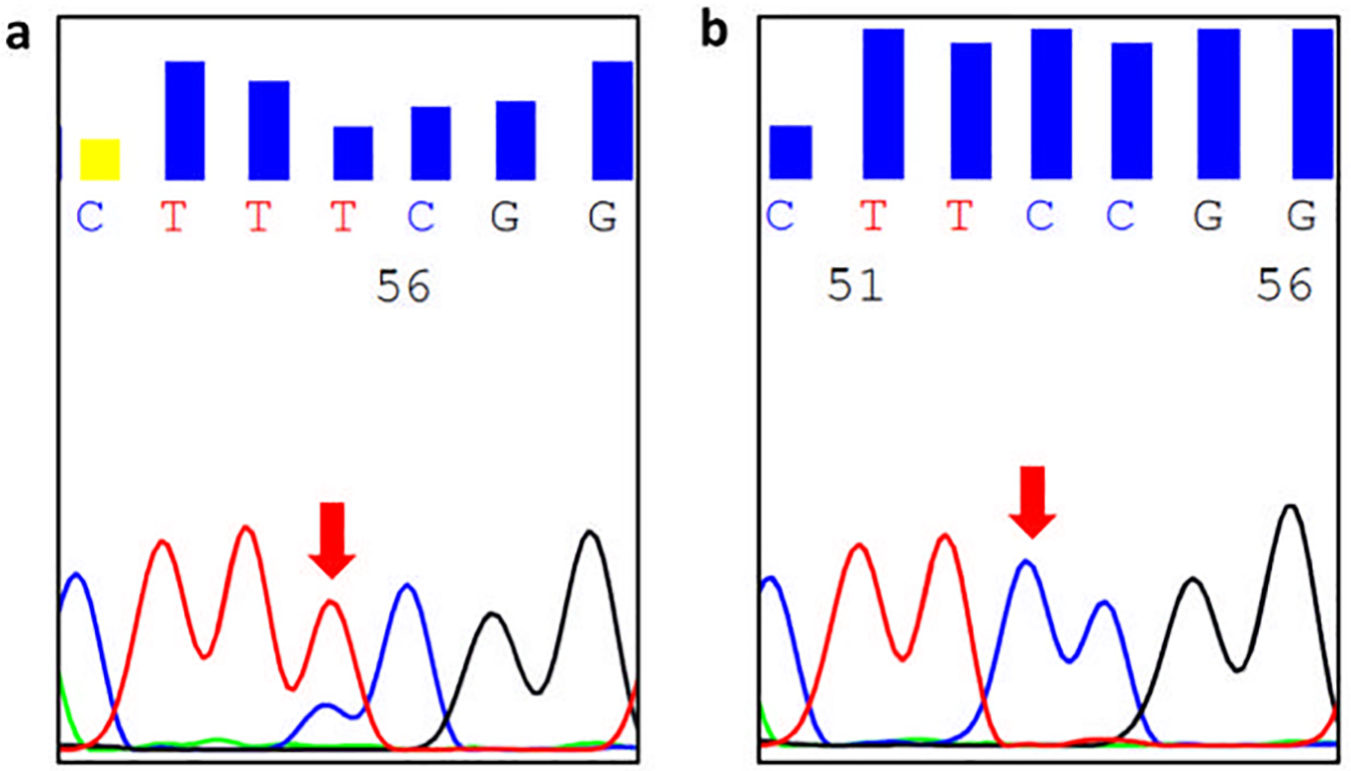
Representative electropherograms of SDHD promoter sequencing from a case with the chr. 11:111,957,523 (TTCC>TTTC) SDHD alteration (a) and a case with wild-type sequence (b).

86 CM were analysed for *SDHD* promoter mutations. We found a chr. 11:111,957,523 (TTCC>TTTC) *SDHD* alteration [one of the mutations reported by Weinhold *et al* [8]] in two CM (2%) (Fig 1). One mutated case was a superficial spreading melanoma diagnosed in 1999, that did not display *BRAF*, *NRAS* and *TERT* promoter mutations, and the patient was alive at the last follow-up (180 months). The other mutated case was an acral melanoma diagnosed in 2009, displayed a -124:G>A *TERT* promoter mutation, but not *a BRAF* mutation; the patient died 24 months after diagnosis, in line with the poor prognosis of CM harbouring *TERT* promoter mutations. [4, 5, 16, 39]. None of the OM cases (26),cutaneous (n = 4) and ocular (n = 7) melanoma cell lines studied harboured any alteration in the promoter of the *SDHD* gene. Due to the lower number of mutated cases, we could not assess any association between the presence of the mutation and the clinico-pathological and prognostic parameters of CM.

### Expression of SDHD protein in CM, OM and melanoma cell lines

**Fig 2:**
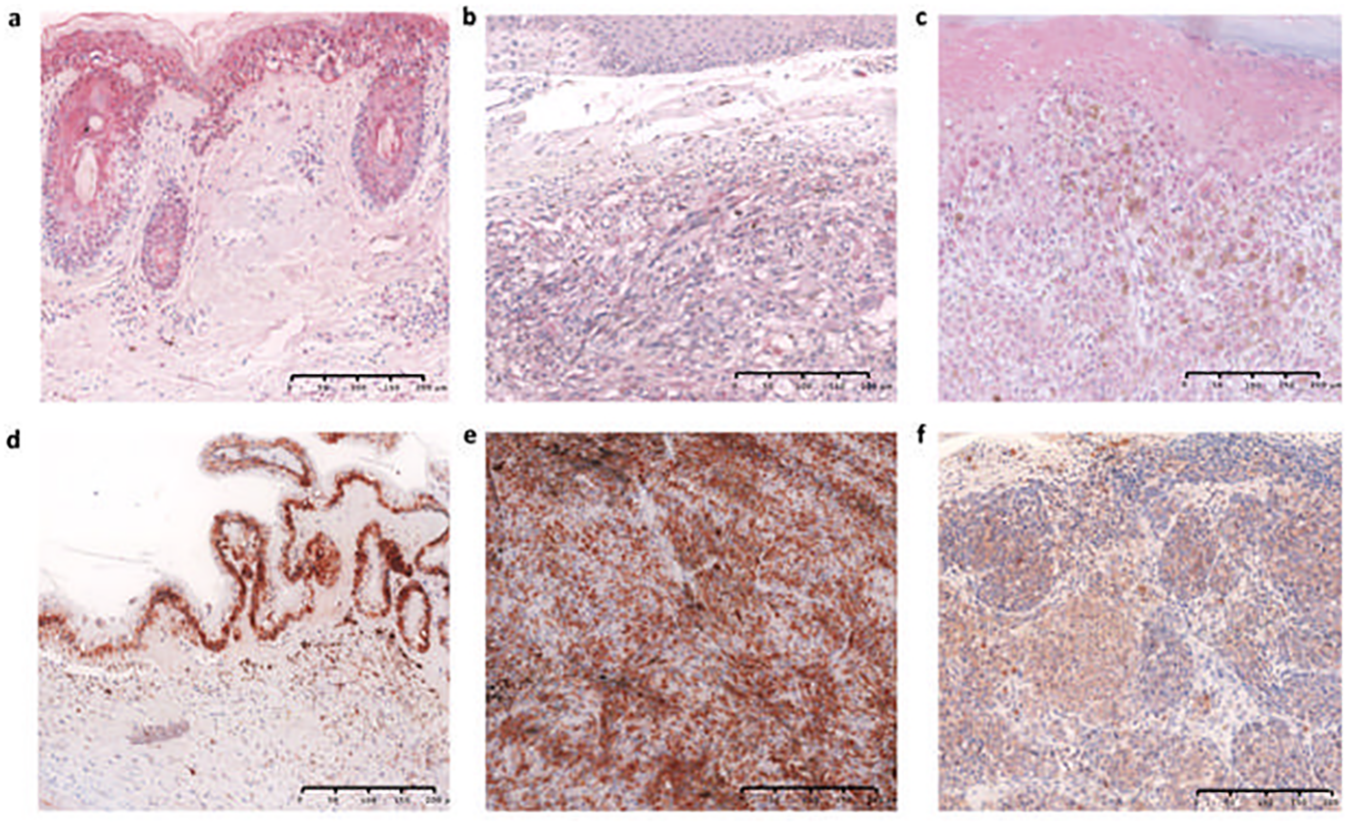
Representative microphotographs of SDHD protein expression in adjacent skin (a), a cutaneous case with the chr. 11:111,957,523 (TTCC>TTTC) SDHD alteration (b), a cutaneous case with wild-type SDHD promoter sequence (c), adjacent ocular structures (d), a uveal case with wild-type SDHD promoter sequence (e) and a conjunctival case with wild-type SDHD promoter sequence (f). APAAP×200 (a-c), HRP×200 (d-f).

SDHD protein expression, evaluated in 107 CM, was cytoplasmic and present in all cases, including those (two cases) with *SDHD* promoter mutations (Fig 2). SDHD was expressed not only in melanocytes/melanoma cells, but also in keratinocytes and in the cells of sebaceous glands and hair follicles. Low staining score (score ≤2) was observed in 41% and moderate/high staining score (score >2) was observed in 59% of melanomas.

SDHD protein expression was also evaluated in 33 OM. Cytoplasmic immunoreactivity was observed in 67% of conjunctival melanomas and 30% of uveal melanoma (Fig 2). SDHD was expressed also in adjacent non-tumour eye structures, mostly iris, retina and ciliary pigment epitheliums. Low staining score (score ≤2) was observed in all the positive conjunctival melanomas and 19% of uveal melanoma, and moderate/high staining score (score >2) was observed in 11% of uveal melanoma.

No association was found between the expression of SDHD and CM histological subtypes or type of sunlight/UV exposure. Concerning the prognostic factors of CM, a significant association was found between lower mean SDHD protein expression and the presence of ulceration (p<0.01) and higher pT stage (p<0.01) (Table 3). No significant association was found between SDHD protein expression and other clinico-pathological parameters (age, sex, mitotic index and tumour thickness), although the same tendency, lower mean SDHD protein expression, was observed with higher thickness and mitotic rate. To evaluate whether SDHD protein expression correlates with disease-free (DFS) and overall (OS) survival in CM, Kaplan Meier curves and univariate Cox regression were performed. No significant association between SDHD protein expression and DFS and OS of the patients was found, although it was observed in the Kaplan Meier curves that patients with low expression of SDHD displayed non-significant reduced DFS and OS compared with patients with high expression of SDHD (S1 Fig). Regarding OM, the low number of conjunctival and uveal cases did not allow the statistical analysis.

**Table 3:** Summary of the statistical associations between SDHD expression and the clinico-pathological parameters of cutaneous melanomas.

Clinico-pathological features	SDHD mean expression level (±SD)	p-value
Pigmentation		
absent	4.37 (3.07)	ns
present	3.74 (2.79)	
Thickness		
≤ 1/mm	4.72 (3.56)	ns
> 1/mm	3.91 (2.78)	
Epidermal ulceration		
absent	4.80 (3.34)	**<0.01**
present	3.11 (2.17)	
Clark level (≤ 1mm) [n (%)]		
I/II	4.05 (3.14)	ns
III/IV	4.21 (3.33)	
Mitotic rate [n (%)]		
**<** 1/mm^2^	4.26 (3.76)	ns
≥ 1/mm^2^	4.18 (2.75)	
pT		
≤ pT2	5.02 (3.63)	**<0.01**
> pT2	3.40 (2.21)	

We also evaluated if the expression of SDHD protein was related to the activation of the MAPK pathway, and the presence of *TERT* promoter mutation and TERT protein expression. No association was found between the expression of SDHD and *BRAF/NRAS* mutations, pERK expression (the readout of MAPK pathway activation) or with *TERT* promoter mutations and TERT protein expression. Due to the low number of mutated cases, we cannot infer if there is a reduction in the protein expression associated with the presence of *SDHD* promoter mutation, but moderate/high staining scores (6 and 9) were found in the two cases with *SDHD* promoter mutations. All the melanoma cell lines studied expressed SDHD protein (S2 Fig).

## Discussion

In this work we established, for the first time, that SDHD protein expression associates with prognostic features of CM.

SDH enzyme has a central role in mitochondrial metabolism [2]. SDHD alterations result in the disruption of the SDH complex and loss of SDH enzymatic activity [40, 41]. In paragangliomas, *SDHB* alterations were linked to malignancy and *SDHD* alterations are more frequent in head-and-neck localized tumours [42, 43]. Recently, *SDHD* promoter mutations were reported in melanomas, associated with reduced gene expression and reduced patient survival [8, 9].

At variance with Weinhold *et al* [8], who reported frequent noncoding alterations in *SDHD* promoter in melanomas, we only found two cases (2%) with *SDHD* alteration in our cutaneous melanoma series, similar to the 4% reported by Scholz *et al* [9]. Several factors may explain these discrepant results when compared with Weinhold *et al* work which was exclusively based on data mining, without sequencing validation, namely the use of different methodologies with different sensitivities, and differences in the cutaneous melanoma subtypes and melanomas staging.

Although C>T alterations are generally considered a marker of UV-exposure, we found a 523C>T *SDHD* mutation in an acral melanoma case that occur in skin without sun exposure, at variance with Scholz *et al* [9] that did not find *SDHD* mutations in acral melanomas. We did not find also any mutations in uveal melanomas, that are considered not associated with exposure to sunlight/UV radiation [19]. No alterations were found by us in cutaneous and ocular melanoma cell lines; these results reinforce the possibility that these alterations are rare in melanomas. The rarity of *SDHD* mutations detected in our series does not allow us to validate the association between the presence of *SDHD* promoter mutation and a reduction in the expression of SDHD gene or the association between this mutation and prognostic parameters of CM, as reported by Weinhold *et al* [8].

SDHD protein was expressed in all melanoma cell lines analysed and in all CM cases, including the two cases with *SDHD* mutation. We found a significant association between low mean SDHD protein expression and the presence of ulceration and high pT stage. Our results indicate an association between the reduction of SDHD protein expression and worst prognosis, but without a significant relation with survival of patients with CM. Although the molecular and cellular mechanisms linking SDH inactivation and tumorigenesis it is not completely understood, SDHD mutation/inactivation results in a loss of electron transport chain complex II activity and in the activation of the hypoxia-angiogenic pathway, namely an increase of EPAS-1, HIF-1 and VEGF expression, which may be the involved mechanism in tumorigenesis [40, 44]. The presence of pigmentation is also linked to increase HIF-1 expression and shorter DFS and OS [45, 46], however in our series, no relation between SDHD expression and pigmentation status was found.

Diverse etiopathogenic mechanisms may operate in the development of cutaneous, conjunctiva and uveal melanomas and it seems that conjunctiva melanomas share more comparable pathogenesis with cutaneous melanomas than with uveal melanomas [47, 48]. We still dispute the biological meaning of absence of SDHD expression in OM, as no *SDHD* promoter mutations were found in the OM cases analysed.

In conclusion, our results indicate that *SDHD* promoter mutation is a rare event in CM and is absent in OM. Yet, SDHD expression might have prognostic relevance in CM. Larger studies are necessary to validate if low expression of SDHD (related or not with the presence of the promoter mutation) might associate with worst prognostic features of CM. Importantly, the metabolic reshape created by SDHD alteration may open a possible therapeutic window that can benefit CM patients, through drugs that can revert this metabolism shift.

## Supporting information

S1 Fig Kaplan Meier curves demonstrating the correlation between SDHD protein expression and disease-free (a) and overall (b) survival of cutaneous melanoma patients. Patients with low expression of SDHD protein (solid line) display reduced disease-free and overall survival compared with patients high expression of SDHD protein (dashed line).
(TIF)

S2 Fig Representative western blot analysis of SDHD protein expression observed in CM and OM melanoma cell lines. All the cell lines analysed express SDHD protein.
(TIF)
