# Supplementary figures and images for: SDHD promoter mutations are rare events in cutaneous melanomas but SDHD protein expression is downregulated in advanced cutaneous melanoma
Source: PLoS One. 2017 Jun 29;12(6):e0180392. doi: 10.1371/journal.pone.0180392 (PMC5491217; doi:10.1371/journal.pone.0180392)

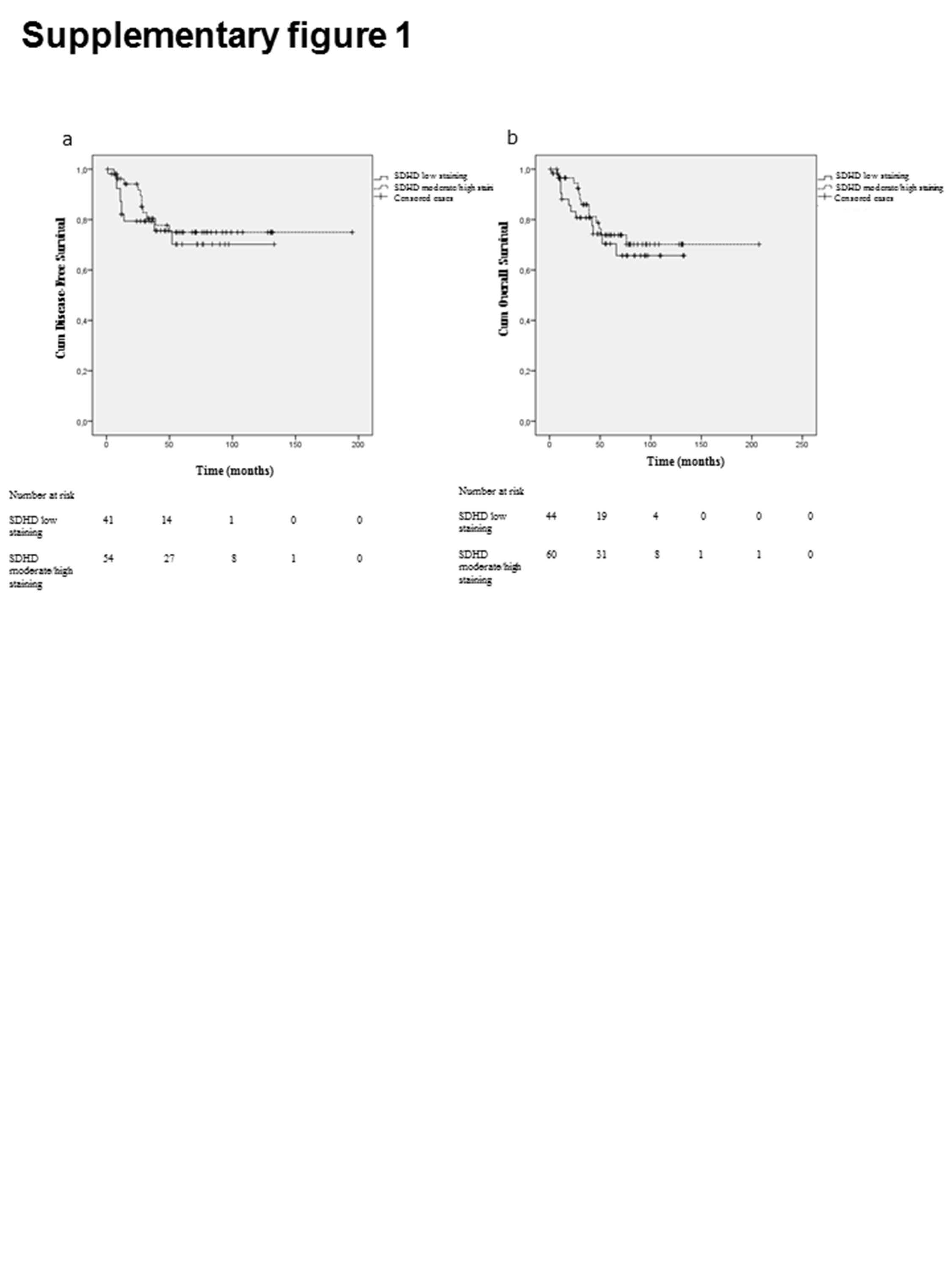

Supplement: S1 Fig — Kaplan Meier curves demonstrating the correlation between SDHD protein expression and disease-free (a) and overall (b) survival of cutaneous melanoma patients. Patients with low expression of SDHD protein (solid line) display reduced disease-free and overall survival compared with patients high expression of SDHD protein (dashed line). (TIF) [file pone.0180392.s001.TIF]

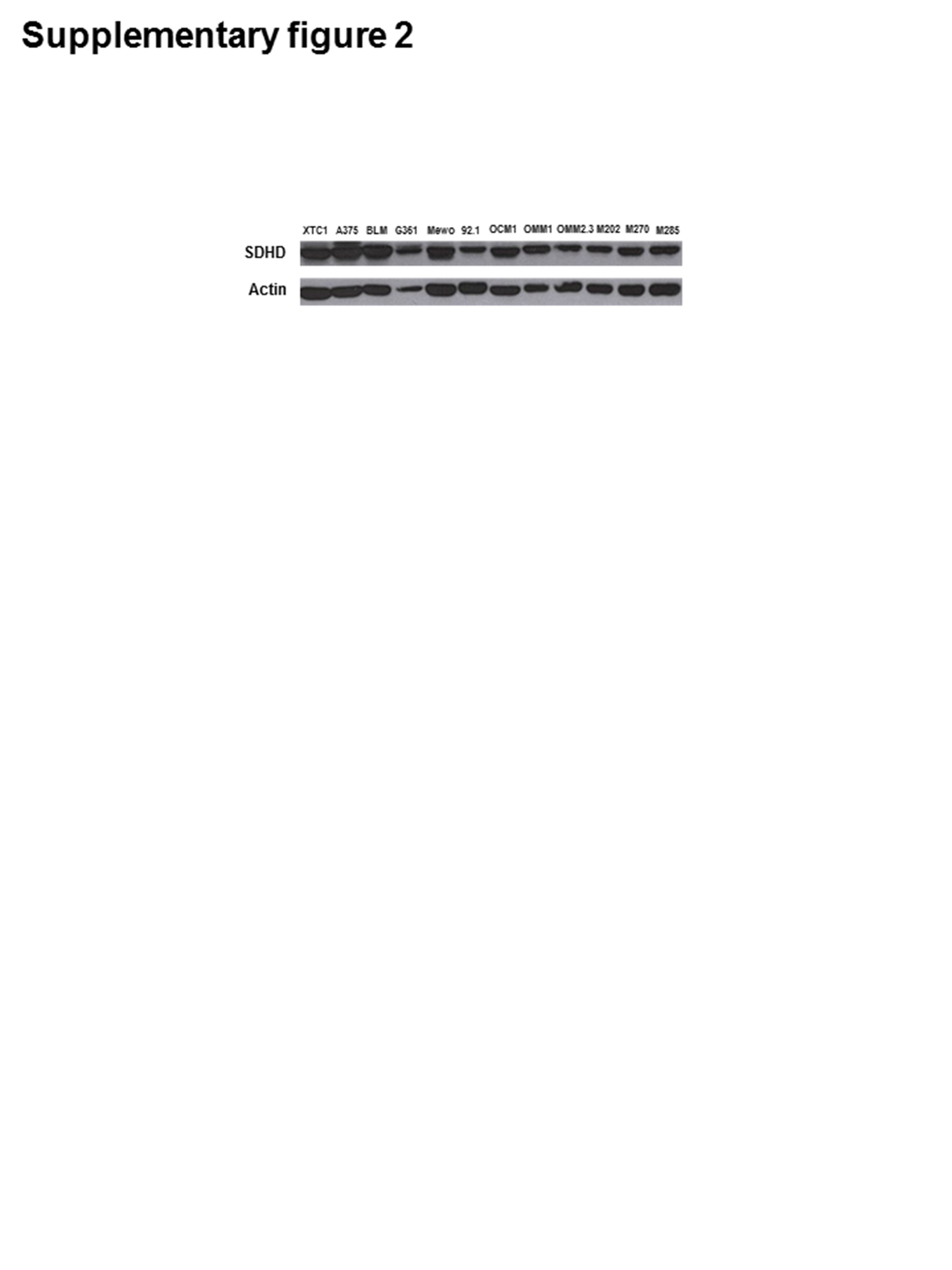

Supplement: S2 Fig — All the cell lines analysed express SDHD protein. (TIF) [file pone.0180392.s002.TIF]
